# Serum macroelements and microelements levels in periparturient dairy cows in relation to fatty liver diseases

**DOI:** 10.1186/s12917-024-04121-9

**Published:** 2024-07-06

**Authors:** Ke-Xin Zhang, Ke Li, Zhe-Hao Li, Xiao-Chen Liu, Meng-Meng Li, Shan Jiang, Rui-Feng Fan, Zhen-Gui Yan

**Affiliations:** 1 College of Veterinary Medicine, Shandong Agricultural University, 61 Daizong Street, Tai’an City, Shandong Province 271018 China; 2 Shandong Provincial Key Laboratory of Animal Biotechnology and Disease Control and Prevention, Shandong Agricultural University, 61 Daizong Street, Tai’an City, Shandong Province 271018 China; 3 Shandong Provincial Engineering Technology Research Center of Animal Disease Control and Prevention, Shandong Agricultural University, 61 Daizong Street, Tai’an City, Shandong Province 271018 China

**Keywords:** Cow, Fatty liver, Macroelements, Microelements, Inductively coupled plasma mass spectrometry, Multivariate analysis

## Abstract

**Background:**

Fatty liver in dairy cows is a common metabolic disease defined by triglyceride (TG) buildup in the hepatocyte. Clinical diagnosis of fatty liver is usually done by liver biopsy, causing considerable economic losses in the dairy industry owing to the lack of more effective diagnostic methods. Therefore, this study aimed to investigate the potential utility of blood biomarkers for the diagnosis and early warning of fatty liver in dairy cows.

**Results:**

A total of twenty-four lactating cows within 28 days after parturition were randomly selected as experimental animals and divided into healthy cows (liver biopsy tested, *n* = 12) and cows with fatty liver (liver biopsy tested, *n* = 12). Inductively coupled plasma mass spectrometry (ICP-MS) was used to determine the macroelements and microelements in the serum of two groups of cows. Compared to healthy cows (C), concentrations of calcium (Ca), potassium (K), magnesium (Mg), strontium (Sr), selenium (Se), manganese (Mn), boron (B) and molybdenum (Mo) were lower and copper (Cu) was higher in fatty liver cows (F). Meanwhile, the observed differences in macroelements and microelements were related to delivery time, with the greatest major disparity between C and F occurring 7 days after delivery. Multivariable analysis was used to test the correlation between nine serum macroelements, microelements and fatty liver. Based on variable importance projection and receiver operating characteristic (ROC) curve analysis, minerals Ca, Se, K, B and Mo were screened as the best diagnostic indicators of fatty liver in postpartum cows.

**Conclusions:**

Our data suggested that serum levels of Ca, K, Mg, Se, B, Mo, Mn, and Sr were lower in F than in C. The most suitable period for an early-warning identification of fatty liver in cows was 7 days after delivery, and Ca, Se, K, B and Mo were the best diagnostic indicators of fatty liver in postpartum cows.

## Introduction

Fatty liver is a common nutritional metabolic disease, which is mainly characterized by an excessive accumulation of lipids in the hepatocytes, and the diagnosis of the disease can currently only be confirmed by liver biopsy [1, 2]. Fatty liver mainly occurs in the first 4 weeks after calving, which is closely related to the negative energy balance of cows in early lactation [3]. Due to negative nutrient balance, numerous fatty acids are produced from adipose tissue to provide the body with energy, which causes an excessive buildup of lipids in the liver [4–6]. These fatty acids have been demonstrated in studies to have negative impact on antioxidant capacity [7]. Meanwhile, numerous macroelements and microelements have been associated with the antioxidant defense system, and a deficiency in any of these nutrients may increase the risk of oxidative stress and metabolic problems in cows [8, 9].

Essential macroelements and microelements have important roles in a variety of physiological processes, particularly energy metabolism and antioxidant defense in cows [10, 11]. For instance, the deprivation of dietary calcium (Ca) eliminated the increase of gluconeogenesis [12]. Magnesium (Mg) is known to be an important macroelements in the homeostatic pathway for regulating blood Ca based on work conducted in cows [13]. Feeding of higher concentrations of dietary Mg can help prevent hypocalcaemia and decreases in plasma free fatty acids concentrations in parturient cows [14, 15]. Meanwhile, potassium (K), Mg, and Ca deficiency have been proven to suppress feed intake and decrease rumen peristalsis [16, 17], aggravating the negative energy balance of cows. However, supplementing with boron (B) decreases lipolysis, and lowers non-esterified fatty acid (NEFA) concentrations [18]. In addition, selenium (Se) consumption can affect lipid metabolism and accumulation in cows [19] and improve oxidative stress and immunity in transition cows [8, 20]. Therefore, timely monitoring of macroelements and microelements deficiencies is required to prevent fatty liver in dairy cows.

Due to the importance of macroelements and microelements in glucolipid metabolism, dynamic monitoring of the content of nine macroelements and microelements in serum in healthy and fatty liver cows after parturition was done using inductively coupled plasma mass spectrometry. This research aimed to ascertain the condition of the metabolism of macroelements and microelements in cows with fatty liver at various time points and determine the most effective macroelements and microelements as diagnostic markers for postpartum fatty liver in dairy cows using statistical analysis, intended to provide some scientific support for clinical prevention and treatment of postpartum fatty liver in cows.

## Results

### Serum parameters and hepatic TG content

**Fig. 1:**
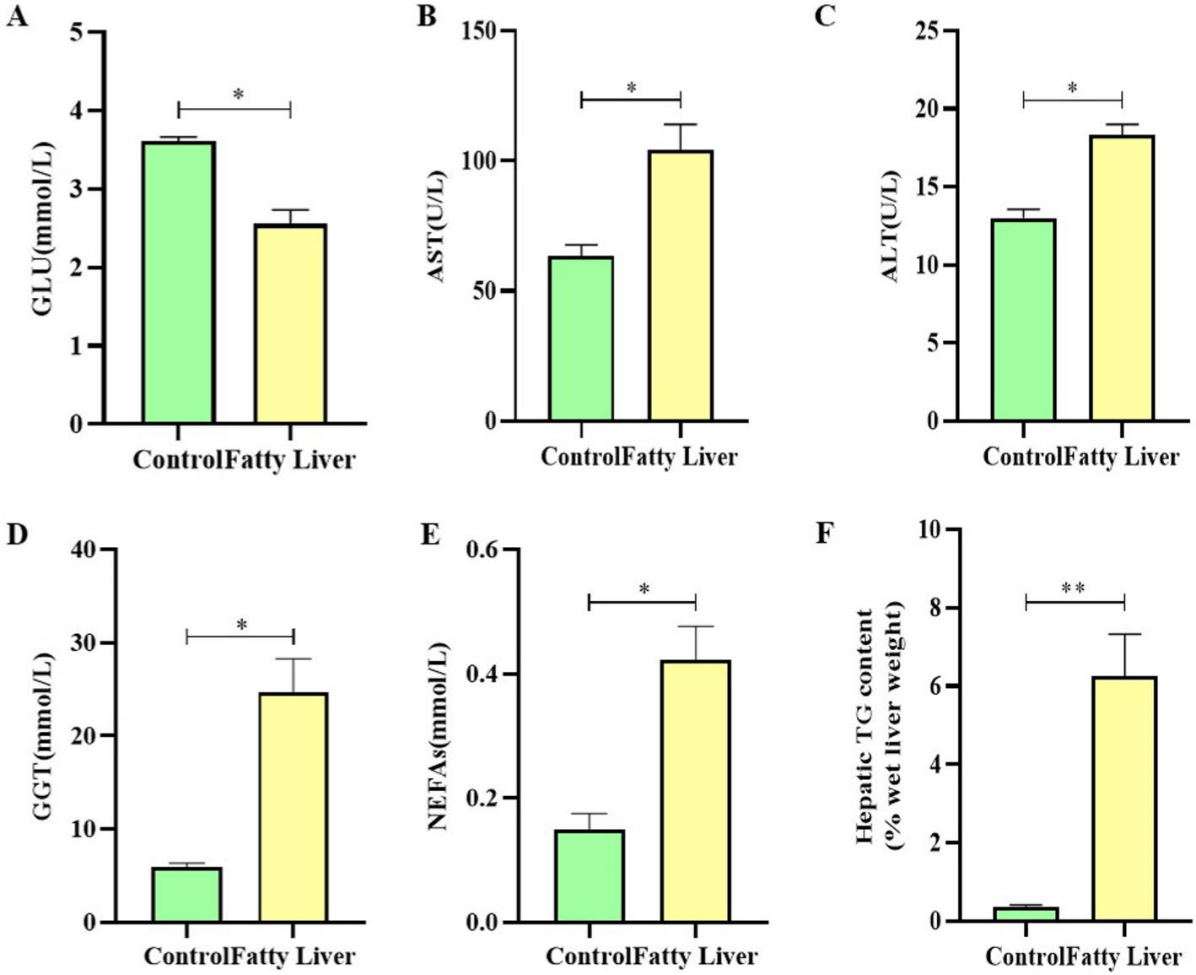
Blood parameters and the indexes of liver tissue. **A** GLU, (**B**) AST, (**C**) ALT, (**D**) GGT, (**E**) NEFA, (**F**) TG in healthy cows (*n*=12) and fatty liver cows (*n*=12). Significant differences were shown as *(*P *< 0.05), **(*P *< 0.01). Values were presented as means±SEM

Serum glucose (GLU) levels were considerably lower in fatty liver cows than in healthy cows (Fig. 1A, *P* < 0.05). Instead, aspartate aminotransferase (AST), alanine aminotransferase (ALT), γ-glutamyl transpeptidase (GGT), and non-esterified fatty acid (NEFA) levels were significantly higher in fatty liver cows than in healthy cows (Fig. 1 B, C, D, E, *P* < 0.05). Meanwhile, fatty liver cows had significantly higher hepatic triglyceride (TG) levels than healthy cows (Fig. 1 F,* P* < 0.01).

### Serum macroelements and microelements content

The serum analysis findings showed that the levels of macroelements and microelements had significant effects on cows with fatty liver (Table 1). Particularly, the serum levels of molybdenum (Mo), B, Se, manganese (Mn), strontium (Sr), Mg, K, and Ca in cows with fatty liver were 42%, 27%, 24%, 20%, 15%, 15%, 9%, and 8% respectively, lower than those in healthy cows. Additionally, serum copper (Cu) levels in fatty liver cows were significantly increased by 20%.

**Table 1:** Serum macroelements and microelements content and comparison between healthy cows and cows with fatty liver

Group	Control (Mean ± SEM)	Fatty liver (Mean ± SEM)	*P* value
Ca(mg/L)	95.13±0.50	87.65±1.16	<0.001
Mg(mg/L)	23.99±0.45	20.43±0.78	0.001
K(mg/L)	178.50±1.88	163.08±2.43	<0.001
Sr(ug/L)	127.52±4.84	108.53±4.14	0.007
Se(ug/L)	94.52±2.74	71.52±3.65	<0.001
Mn(ug/L)	45.97±2.10	36.67±2.93	0.017
B(ug/L)	238.27±11.05	173.08±9.62	<0.001
Cu(ug/L)	661.62±17.32	826.25±35.17	0.001
Mo(ug/L)	9.90±0.76	5.70±0.52	<0.001

### Fluctuations in serum macroelements and microelements levels

**Fig. 2:**
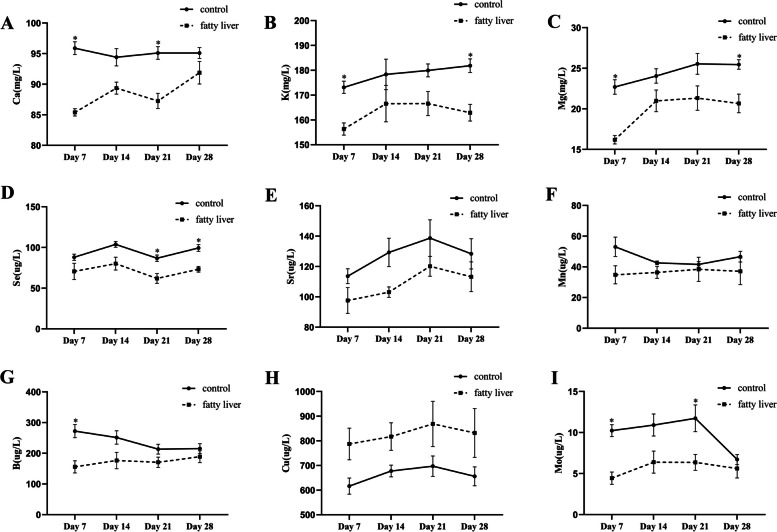
Serum macroelements and microelements content dynamics in cows after parturition. **A** Ca, (**B**) K, (**C**) Mg, (**D**) Se, (**E**) Sr, (**F**) Mn, (**G**) B, (**H**) Cu, (**I**) Mo. Significant differences were shown as *(*P* < 0.05), **(*P *< 0.01). Values were presented as means±SEM

The relationship between the observed variations in the amounts of macroelements and microelements and the time of lactation in both healthy and cows with fatty liver was shown in Fig. 2. We found that the serum levels of Ca, K, Mg, Sr, Se, Mn, B, and Mo notably fluctuated during lactation in both healthy and cows with fatty liver. Especially on day 7 after calving, significant changes in macroelements and microelements were observed.

### Correlation analysis

**Fig. 3:**
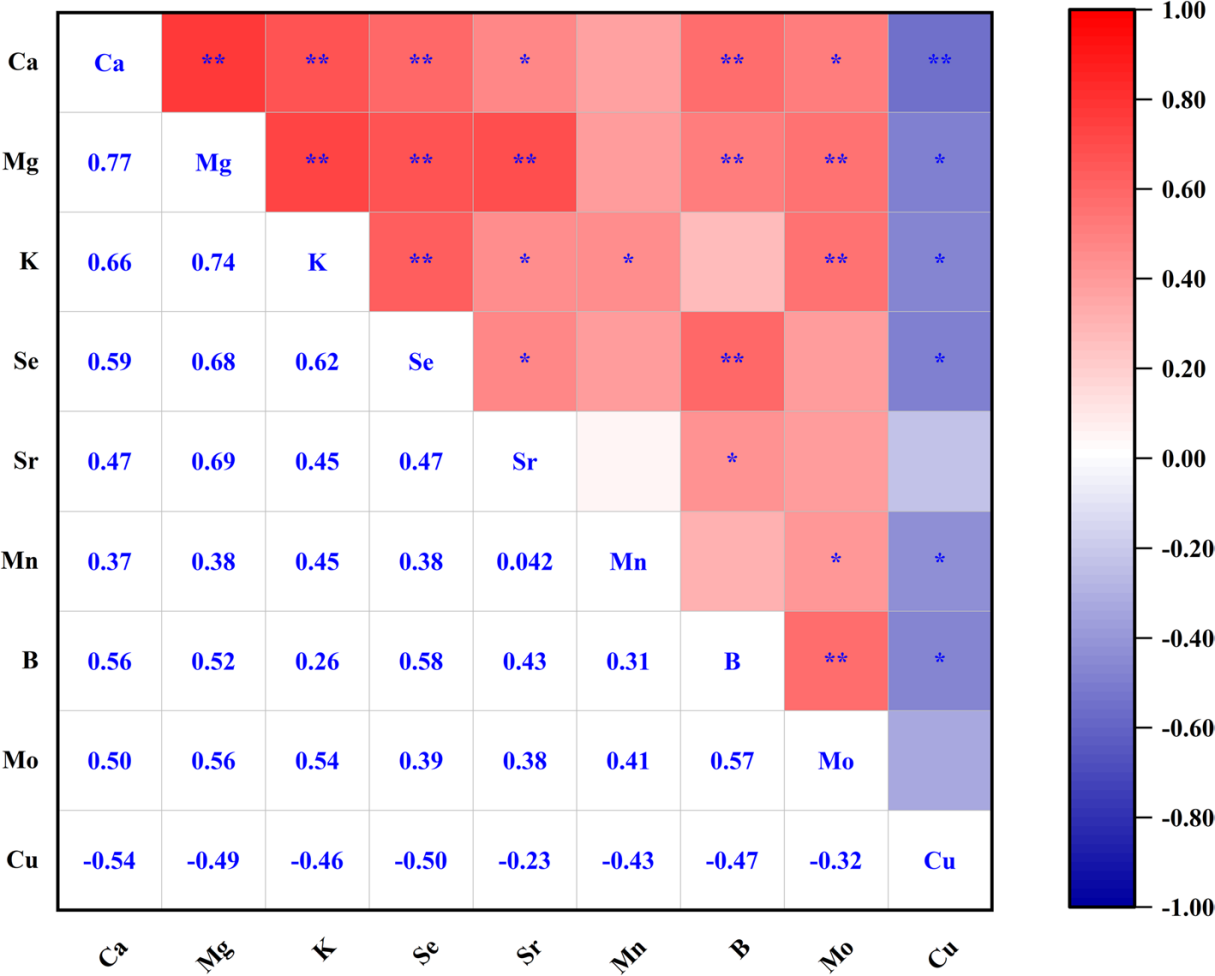
Heat map of Pearson’s correlation among macroelements and microelements content present. Significant differences were shown as *(*P *< 0.05), **(*P* < 0.01)

To investigate the dependency of the components in the serum, Pearson's correlation analysis was used. The discoveries of the correlation matrix are shown in Fig. 3. According to Pearson's correlation coefficient, an absolute value > 0.5 showed a good or strong correlation, and an absolute value < 0.5 indicated a weak correlation. Most minerals had significant positive correlations with each other while having a negative correlation in Cu (Fig. 3). The correlations among Ca-Mg, Ca-K, Ca-Se, Ca-B, Ca-Mo, Mg–K, Mg-Se, Mg-Sr, Mg-B, Mg-Mo, K-Se, K-Mo, Se-B, Mo-B, Cu-Ca, and Cu-Se were stronger (*P* < 0.05).

### Multivariate analysis

**Fig. 4:**
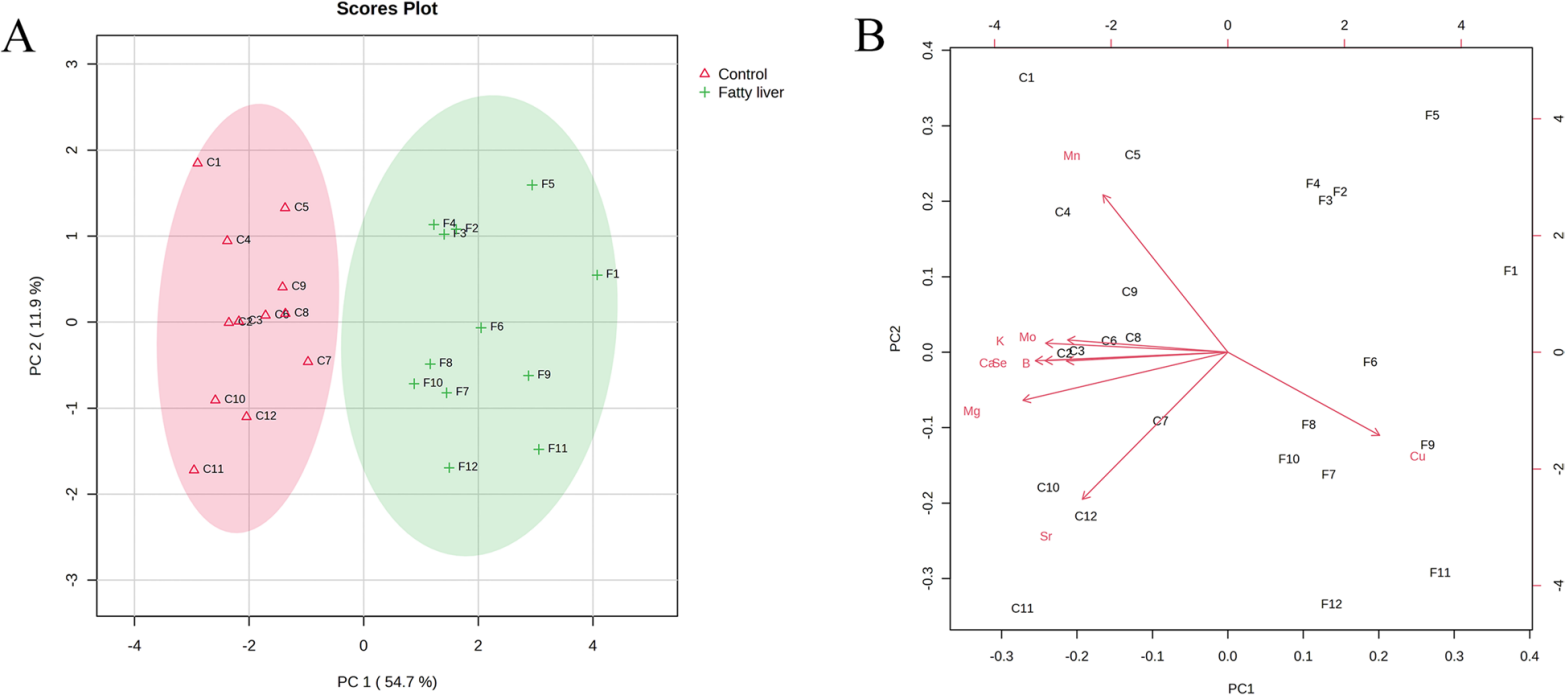
Differential macroelements and microelements of healthy cows and cows with fatty liver. **A** PCA score plot dependent on macroelements and microelements of healthy cows and cows with fatty liver. **B** The principal components analysis's loading plot of the investigated variables

The data of the study sample were subjected to principal component analysis (PCA) to improve the visualization of the intrinsic differences between the two groups. The following minerals are used by the resulting groupings as predictors: Ca, Mg, K, Se, Sr, Mn, B, Mo, and Cu. A distinct tendency of separation between healthy cows and fatty liver cows was seen in the score plot results (Fig. 4A). In particular, the first two principal components (PC1 and PC2) accounted for 66.6% of the total variance, with PC1 contributing 54.7% and PC2 contributing 11.9%. The principal component analysis's depicted loading plot of the investigated traits revealed that associated features were positioned on the plot with close distances, which was consistent with correlation analysis (Fig. 4B). For instance, only Cu was found on the right side of the plot, whereas factors like Ca, K, Mo, Se, B, Mg, Mn, and Sr that had a negative impact on the health of cattle were found on the left.

**Fig. 5:**
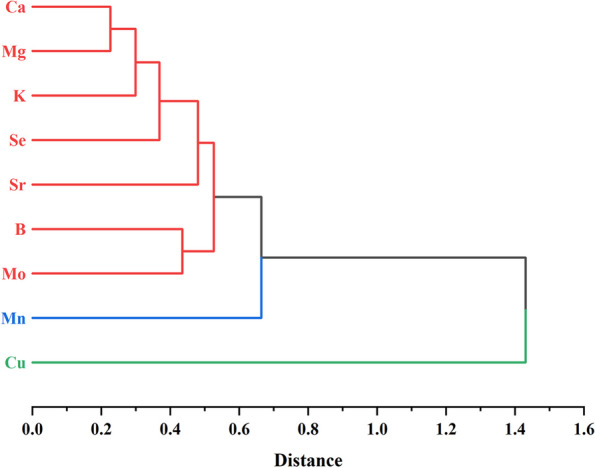
Hierarchical cluster analysis (HCA) tree diagram

The researched characteristics were divided in a manner similar to the PCA plot depending on the resulting dendrogram using clustering analysis (Fig. 5), with Ca, Mg, K, Se, Sr, Mn, B, and Mo clustered together, while Cu was classified separately. The result could further substantiate the classification of samples by the PCA model.

**Fig. 6:**
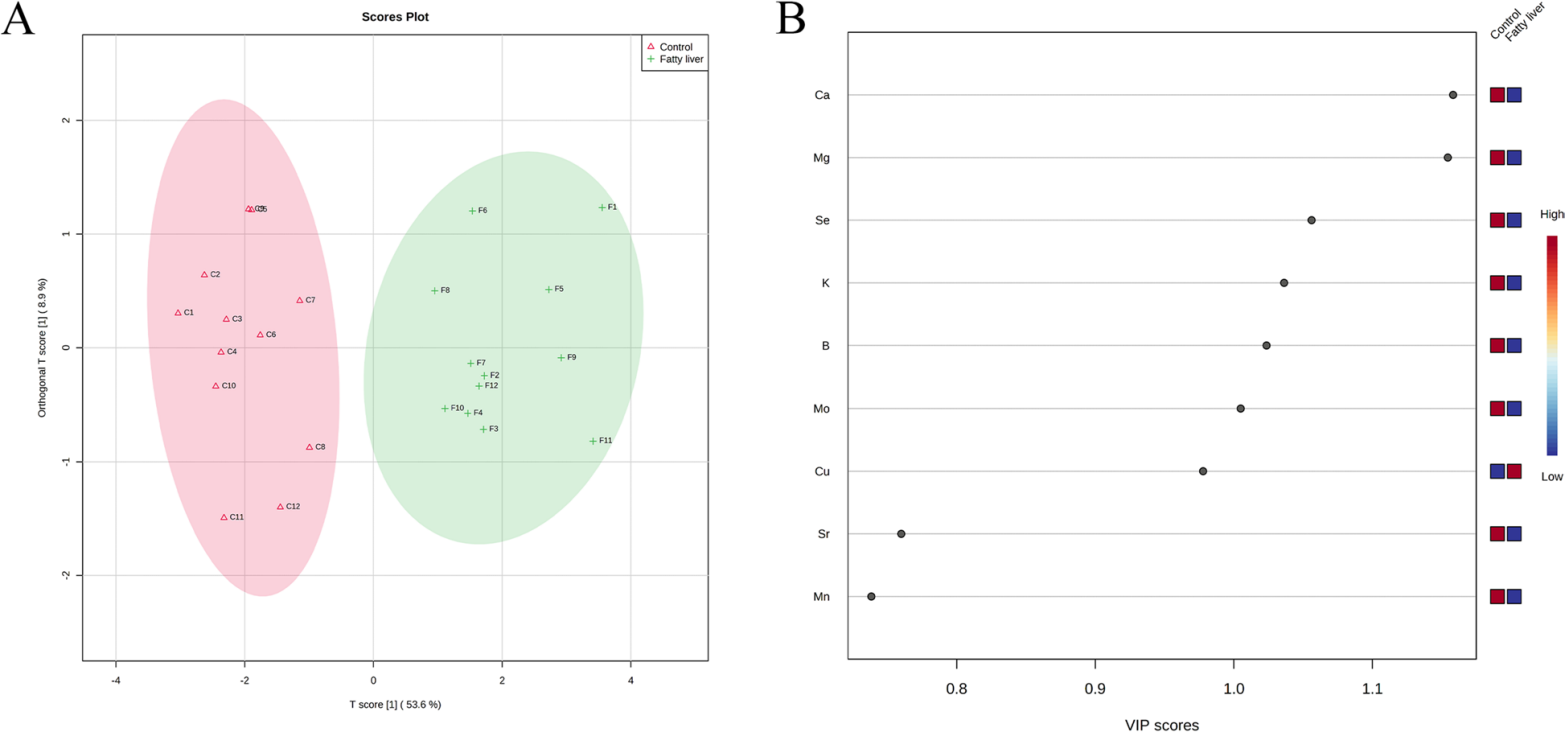
Orthogonal partial least squares discriminant analysis (OPLS-DA). **A** OPLS-DA score graph. The red triangle represents the healthy group and the green cross represents the fatty liver group. **B** The VIP score graph of OPLS-DA

The predictors utilized were the same as those in the final PCA model. The orthogonal partial least squares discriminant analysis (OPLS-DA) figure showed distinct divisions between healthy (red) cows and fatty liver (green) cows (Fig. 6A). In addition, variable importance values were indicated in Fig. 6B, with six variables (Ca, Mg, Se, K, B, and Mo) having higher variable importance for projection (VIP) values (> 1.0). It indicated that these variables were important indicators to distinguish fatty liver cows.

### ROC curve analysis

**Fig. 7:**
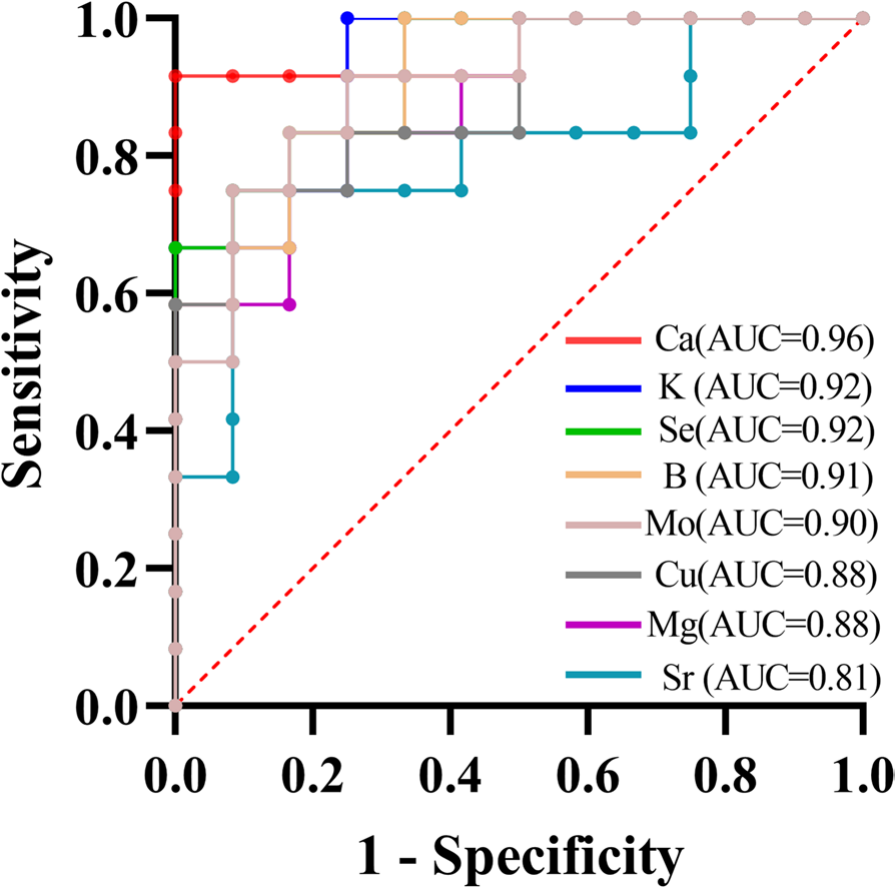
ROC curve analysis of macroelements and microelements

The ROC curve analysis was used to determine the potential value of various macroelements and microelements for fatty liver disease (Fig. 7). Macroelements and microelements with AUC values approaching 1 had a significantly improved predictive power as biomarkers. The experimental results revealed that five minerals (Ca, K, Se, B and Mo) had an area under the curve (AUC) of > 0.9 (Fig. 7). The sensitivity, specificity, and AUC values for the serum calcium levels predictive capacity were 0.92, 1.00, and 0.96 respectively. Furthermore, the AUC of 0.92 with a sensitivity of 1.00 and specificity of 0.67 was obtained for the serum Se. Meanwhile, the serum levels of K showed a sensitivity of 1.00, specificity of 0.75, and the AUC of 0.92. Moreover, the AUC of 0.91 was calculated for serum B levels with a sensitivity of 1.000 and specificity of 0.67. Finally, the serum levels of Mo showed a sensitivity of 0.92, specificity of 0.75, and the AUC of 0.90 (Table 2).

**Table 2:** The sensitivity and specificity of macroelements and microelements in dairy cows

Parameters	Sensitivity	Specificity	*P* Value
Ca	0.92	1.00	<0.01
Se	1.00	0.67	<0.01
K	1.00	0.75	<0.01
B	1.00	0.67	<0.01
Mo	0.92	0.75	<0.01

## Discussion

Fatty liver is a prevalent disease in dairy cows during lactation. In order to minimize the influence of other variables on clinical laboratory markers, cows with comparable parity, body condition scores, and ages were selected for the experiment. We first analyzed the amount of macroelements and microelements in the serum by ICP-MS and found that the concentrations of Ca, K, Mg, Sr, Se, Mn, B, and Mo were considerably lower than in healthy cows, although the amounts of Cu were significantly higher. Meanwhile, the concentrations of the above macroelements and microelements fluctuated significantly from delivery to 28 days. In addition, after visualization of the collected data using PCA and OPLS-DA algorithm, the trend of separation between groups was found to be obvious. Simultaneously, there were strong connections between the elements Ca, K, Mg, Sr, Se, Mn, B, and Mo. Through ROC curve analysis and variable importance values, the results further revealed the biological importance of Ca, Se, K, B and Mo elements in the diagnosis of fatty liver in cows.

Both group comparisons and multiple regression analysis demonstrated that the occurrence of fatty liver in cows may be associated with the deficiency of Ca, K, Mg, Mo, B, Se, Sr, and Mn. In agreement with Patel et al. and Fiore et al., our data obtained showed that cows with fatty livers had elevated serum levels of NEFA and GGT and decreased blood GLU, and the changes may have led to disturbed glucolipid metabolism and oxidative stress in cows [2, 21, 22]. Disorders of glucolipid metabolism were linked to aberrant macroelements and microelements metabolism, according to studies done on people and animals [23, 24]. There was still a lack of clarity regarding the molecular processes of Mn in the etiology of fatty liver. However, a cohort research found that having high blood Mn levels may serve as a potential defense against fatty liver [25, 26]. In addition, increased triglyceride levels were associated with lower blood Ca and Mg concentrations [27–29]. Supplementing with B and Mo had been shown to lessen the amount of fat that build up in the liver and lowered the risk of fatty liver [30–32]. Dairy cows in the transition period usually had negative energy balance, which was influenced by the amount of intake the cow received [33]. It was worth noting that blood Ca and K concentrations in feed can affect the dry matter intake of cows, and supplementation with Ca and K can alleviate the extent of negative energy balance in cows [34–36]. So far, no uniform conclusion has been reached on the relationship between macroelements and microelements content and fatty liver, but our experimental data and the above report suggested that changes in macroelements and microelements content may have contributed at least to some extent to the development of fatty liver in dairy cows.

Fatty acids increased sharply in the serum and liver of cows after parturition and were metabolized to provide energy. However, high concentrations of fatty acids also showed lipotoxicity in liver tissue. Fatty acid-induced hepatic lipotoxicity was reduced by improving mitochondrial function, lowering reactive oxygen species (ROS) levels and increasing fatty acid oxidation [37]. As macroelements and microelements are the basis for mitochondrial function and lipid metabolism [38, 39]. Macroelements and microelements deficiencies can lead to mitochondrial dysfunction and oxidative stress. Previous studies had demonstrated the existence of mitochondrial dysfunction in cattle with fatty liver [40]. However, studies in the metabolism of macroelements and microelements in the serum of dairy cows with fatty liver were relatively limited. Data from mice have demonstrated that deep seawater containing Mg, Ca and K can enhance the antioxidant system and inhibit fatty acid biosynthesis in mice, with the strongest preventive effect on fatty liver [41]. Meanwhile, it has been demonstrated that the addition of macroelements and microelements to the diet can improve cows' antioxidant status [42, 43]. Specifically, Se and B supplementation can increase the levels of antioxidant enzymes, reducing the incidence of fatty liver and other metabolic diseases [44, 45]. Moreover, Mn and Cu are also necessary parts of the superoxide dismutase (MnSOD, Cu/Zn SOD), and supplement of the above minerals can decrease mitochondrial oxidative stress and improve the capacity of ROS to be scavenged [46, 47]. Meanwhile, Cu also promotes mitochondrial biogenesis and fatty acid oxidation through the regulation of AMP-activated protein kinase activity, improving the development of fatty liver [48]. In our study, serum copper levels were significantly higher in cows with fatty liver than in healthy cows, which may be related to the mobilisation of copper by the organism in response to oxidative damage. Furthermore, Cu metabolism levels in cows were highly susceptible to Mo levels [49]. Mo deficiency promoted the absorption of Cu, which may also lead to elevated levels of Cu. Moreover, fatty acid overload and intrahepatic lipid accumulation induced endoplasmic reticulum stress, which was associated with reduced Sr levels [50]. Sr deficiency was observed in cows with fatty liver in the present study.

In summary, macroelements and microelements are critical for lipid oxidation and oxidative stress resistance. Oxidative stress and disturbed lipid metabolism are present in cows with fatty liver and play a key role in hepatic lipid accumulation currently confirmed by in vivo studies [40]. Therefore, we suggest that deficiency of Ca, K, Mg, Se, B, Mo, Mn, and Sr may contribute to the occurrence of fatty liver in cows.

## Conclusion

This study found that Ca, K, Se, B, Mo, Mn, Mg, and Sr were severely lacking in postpartum fatty liver cows, but Cu content was dramatically raised. The differences between healthy and fatty liver cows were most pronounced at 7 days after parturition. In conclusion, macroelements and microelements imbalances may be one of the key factors in fatty liver disease.

## Materials and methods

### Experimental animals

The present study protocol was approved by the Ethics Committee on the Care and Use of Laboratory Animals at Shandong Agricultural University (Tai’an, China) (Number: SDAUA-2019–057). The third edition of the "Guide for the Care and Use of Agricultural Animals in Research and Teaching" contained the fundamentals and recommendations for providing humane care to the animals [51]. The cows used in this study were selected from a dairy farm in Zaozhuang, Shandong, China. All cows were housed in freestalls, milked twice daily, and with ad libitum access to tap water. Every cow was fed the same way and with the same fundamental diet formula (Table 3). We randomly selected postpartum Holstein cows for a basic body condition examination and excluded the effects of other major diseases (such as ulcer hoof disease, afterbirth retention and mastitis). TG level in the liver was a common standard for diagnosing fatty liver in cows. Healthy cows had TG concentration of below one percent of the wet weight of the liver tissue; fatty liver cows were seen in those whose TG amount was greater than 1% of the wet weight of the liver tissue [52]. Healthy cows (*n* = 12) and cows with fatty liver (*n* = 12) were selected for the experimental study. Table 4 displayed the basic body features of healthy cows and cows with fatty liver.

**Table 3:** Nutritional composition of the diet of dairy cows

Components	Percentage (%)
Silage	40.3
Mixes ^1^	23.5
Soybean cake	12.5
Clover	10.1
Tablet corn	5.1
Cottonseed	3.4
Molasses	2.7
Beet pulp	2.2
Fat powder	0.2

Mixes^1^: corn 50%, DDGS 12%, bran 8%, soybean meal 19.7%, rooibos 5%, baking soda 3%, magnesium chloride 1%, probiotics 1%, calcium 0.3%

**Table 4:** Description of basic information of fatty liver cows (*n*=12) and healthy cows (*n*=12)

Variables	Control (Mean ± SEM)	Fatty liver (Mean ± SEM)
Fetuses	2.67 ± 0.40	2.42 ± 0.34
Age (mon)	50.00 ± 4.82	50.33 ± 5.83
Physical condition	3.09 ± 0.12	3.04 ± 0.08

### Sample collection

Blood samples were collected from the caudal vein within 1 h of the morning feeding on days 7, 14, 21, and 28 after partutition. The serum was subsequently prepared via centrifugation (2500 r/min) and stored at -80 °C.

Hepatic tissue samples were removed from the right side of the cow between the 11–12 ribs within 14 d after parturition with a liver biopsy needle. Briefly, then the hair between the ribs was removed with a razor, and the skin of the puncture area was disinfected with 75% alcohol and povidone-iodine. After that, local anesthesia was performed with 5% lidocaine hydrochloride by subcutaneous injection, then a stabbing incision of about 1 cm was made on the skin with a 22# scalpel, and the liver tissue was removed through the puncture device, washed with 0.9% saline, and stored frozen in a liquid nitrogen tank.

### Determination of blood biomarkers

An automatic biochemical analytical system (Hitachi 7020, Tokyo, Japan) was used to measure the concentrations of the biochemical indexes for GLU, AST, and ALT in serum (GLU: GL3815; AST: AS3804; ALT: AL3801, Randox Laboratories, Crumlin, UK). The serum levels of NEFA and GGT in liver tissues were measured with reagent kits (NEFA: A042-2–1; GGT: C017-2–1, NanJingJianCheng, Nanjing, China).

### Determination of TG content

TG reagent kit was used for the determination of TG (TG: A110-1–1, NanJingJianCheng, Nanjing, China). The liver tissue samples and saline (0.9%) were mixed with a weight (g): volume (mL) ratio of 1:9. The liver tissue solution was mechanically homogenized for 10 min (2500 r/min), and the supernatant was collected for TG analysis. Total protein concentration was determined by BCA method (A045-4, NanJingJianCheng, Nanjing, China). Cows were designated fatty liver (*n* = 12) or healthy (*n* = 12) according to the criteria for determining fatty liver.

### Evaluation of serum macroelements and microelements Levels

5 mL of nitric acid was added to the serum for sample digestion using the microwave digestion method. The contents of Ca, K, Mg, B, Se, Sr, Mo, Cu, and Mn in serum samples were analyzed by Agilent 7800 ICP-MS (Agilent Technologies, Tokyo, Japan). Diluted calibration solution was prepared with calibration standards. Specific concentrations of Sc, Ge, Rh, In, Bi were added to all calibration solutions and samples as internal standards. The basic validation of the parameters included the recognition of recovery, ranging from 90 to 110%. The ICP-MS system summary operating characteristics were in Table 5.

**Table 5:** Characteristics of ICP-MS system operation

Name of the parameter	Parameters
RF Power	1500W
Plasma gas flow rate	15 L/min
Carrier gas flow rate	0.80L/min
Auxiliary airflow	0.40L/min
Helium flow rate	4 - 5 mL/min
Nebulization room temperature	2°C
Sample lift speed	0.3 r/s
Atomization	High Salinity/Concentric Nebulizer
Sampling cone/Interception	Nickel/Platinum Cone
Sampling depth	8 -10mm
Measurement points per peak	1-3
Number of repetitions	2-3

### Statistical analysis

SPSS software (SPSS 13.0 software, SPSS Inc., Chicago, IL) was used to analyze the data, and the results were expressed as the means ± SEM. The Shapiro–Wilk test was used to measure the distribution of the variables under study, and additional statistical analyses were performed based on the results. The student’s t-test and the Wilcoxon test were used for statistical evaluation of normally and non-normally distributed variables, respectively. The correlation between the nine macroelements and microelements (Ca, K, Mg, Se, B, Mo, Mn, Sr, and Cu) was evaluated using Pearson correlation analysis. The MetaboAnalyst 5.0 online software was used to perform PCA, OPLS-DA, and HCA. *P* values of 0.05 or below were regarded as statistically significant. Finally, ROC curve analyses were drawn using GraphPad Prism (GraphPad 8.0 Software, San Diego, CA, USA) to assess the best diagnostic markers for fatty liver in dairy cows.

## Data Availability

The data used to support the findings of this study are available from the corresponding author upon request.

## Abbreviations

ALT: Alanine aminotransferase
AST: Aspartate aminotransferase
B: Boron
Ca: Calcium
Cu: Copper
GGT: γ-Glutamyl transpeptidase
GLU: Glucose
HCA: Hierarchical cluster analysis
ICP-MS: Inductively coupled plasma mass spectrometry
K: Potassium
Mg: Magnesium
Mn: Manganese
Mo: Molybdenum
NEFA: Non-esterified fatty acid
OPLS-DA: Orthogonal partial least squares-discriminant analysis
PCA: Principal component analysis
ROC: Receiver operating characteristic
Se: Selenium
Sr: Strontium
